# New awakenings: current understanding of sleep dysfunction and its treatment in Parkinson’s disease

**DOI:** 10.1007/s00415-019-09651-z

**Published:** 2019-12-05

**Authors:** Lindsay H. M. Keir, David P. Breen

**Affiliations:** 1 Department of Clinical Neurosciences, Western General Hospital, Edinburgh, UK; 2 Centre for Clinical Brain Sciences, University of Edinburgh, Room FU303g, Chancellor’s Building, 49 Little France Crescent, Edinburgh, E16 4SB UK; 3 Anne Rowling Regenerative Neurology Clinic, University of Edinburgh, Edinburgh, UK; 4 Usher Institute of Population Health Sciences and Informatics, University of Edinburgh, Edinburgh, UK

**Keywords:** Sleep, Circadian, Parkinson’s, Treatment, Glymphatic

## Abstract

The non-motor features of Parkinson’s disease (PD) are increasingly being recognised. This review deals with the spectrum of sleep disorders associated with PD, which have a multifactorial aetiology and can significantly have an impact on the quality of life of patients and their carers. Some sleep disorders represent a prodromal phase of PD, with REM sleep behaviour disorder (RBD) being of particular interest in this regard, whereas others become more common as the disease advances. Understanding the pathophysiology of these sleep disturbances will hopefully lead to new treatment opportunities in the future. The recent discovery of the glymphatic system for removal of waste products from the brain has also raised the possibility that sleep disruption may cause or accelerate the underlying disease process.

## Introduction

Parkinson’s disease (PD) affects 1–2% of adults aged over 65 years and is the second most common degenerative brain disorder. It has long been diagnosed based on its cardinal motor features: bradykinesia, rigidity and tremor. More recently, the prevalence and extent of non-motor features in PD has come to be recognised. These non-motor manifestations are varied and include dementia, mood disorders, autonomic disturbances, pain and sleep dysfunction [54]. Some of these non-motor features can appear prior to PD motor features. They can have marked effects on quality of life and even patient safety (such as increased risk of injury and accidents due to cognitive problems and excessive daytime sleepiness) [10, 42]_._ The previous under-appreciation of non-motor features in PD was probably a combination of under-reporting of symptoms from patients, as well as a lack of awareness from their physicians [18].

In the PRIAMO study, Barone et al. found sleep disorders to be the second most prevalent non-motor complaint in a large cohort of 1072 PD patients, affecting 64% of patients across the entire disease spectrum [3].

This review will discuss the sleep disturbances associated with PD, with particular emphasis on management options and research priorities. It is not intended to be an exhaustive review on the topic [49, 64]. Instead, we will concentrate on topics where there have been recent advances that may lead to an improved understanding or new therapeutic approach to sleep dysfunction in PD.

## The anatomy and function of sleep

Physiologically, sleep can be divided into rapid eye movement (REM) and non-rapid eye movement (NREM) sleep. NREM constitutes the majority of sleep and can be further sub-divided into three stages (N1, N2 and N3), with depth of sleep and its restorative function increasing from N1 to N3. The remainder of the night is accounted for by REM sleep. The stages run cyclically throughout the night, with blocks of slow wave sleep getting shorter as the night goes on. The proportion of slow wave sleep reduces with ageing.

The body’s control of sleep is dependent on the delicate balance between sleep–wake homeostasis and circadian rhythms. The suprachiasmatic nucleus (SCN), a brain region located in the anterior hypothalamus, acts as the body’s master clock and coordinates a robust 24-h rhythm that incorporates sleep and other key bodily functions. In turn, the SCN receives input from external stimuli (such as light cues, motor activity and feeding patterns) and exerts its influence through a variety of neural and hormonal mechanisms. “Clock genes” are vital components within the cellular machinery that drive the circadian signal in every cell in the body via auto-regulatory feedback loops [63].

There are a number of theories regarding why we sleep: to enable cellular processes to recover from the day (e.g. oxidative stress), to facilitate the clearance of waste proteins from the brain, and to optimise learning and memory. Initially postulated by Tononi and Cirelli, the ‘synaptic homeostasis hypothesis’ proposes that sleep promotes synaptic downscaling in the brain following the strengthening of synaptic connections during wakefulness in response to new information [60]. Studies have since highlighted the complexity of this system, with various brain regions, sleep stages and genes being implicated. In 2017, Li et al. [36] highlighted the importance of REM sleep on synaptic spine formation and pruning. The role of sleep in synaptic plasticity and memory consolidation has been reviewed elsewhere [52].

## Spectrum of sleep disorders in PD

Sleep disorders in PD encompass insomnia (difficulties with sleep initiation and/or sleep maintenance), parasomnias [particularly REM sleep behaviour disorder (RBD)], excessive daytime sleepiness (EDS), periodic limb movements of sleep (PLMS), restless leg syndrome (RLS) and obstructive sleep apnoea (OSA). These disorders can be directly related to underlying disease pathology, secondary to symptoms of the disease, or secondary to medication side effects.

Up to 90% of PD patients experience some form of sleep dysfunction [39]. Studies have used a variety of validated sleep scales to demonstrate sleep problems in PD patients: Parkinson’s Disease Sleep Scale 2 (PDSS-2), Scales for Outcomes in Parkinson’s Disease–Sleep (SCOPA-SLEEP), Pittsburgh Sleep Quality Index (PSQI) and Epworth Sleepiness Scale (ESS). Most of these scales evaluate symptoms in the recent past (over the prior 1–4 weeks), with questions designed to probe different PD symptoms that might influence sleep. In doing so, these questionnaires not only assess the presence and severity of sleep problems but can also help to identify their cause and point towards suitable management strategies. The advantages and disadvantages of specific sleep questionnaires, both generic and PD-specific ones, have been reviewed elsewhere [34].

Peeraully et al. performed a systematic review on polysomnography case–control studies in PD [48]. Compared to controls, PD patients typically reported an increased number of nocturnal awakenings (classified as a period of wakefulness of over 15 s) and had shorter sleep time. A number of other studies have shown reduced sleep efficiency, increased sleep latency and reduced REM sleep, but the data are more heterogeneous.

EDS is a feeling of sleepiness that impairs alertness and the ability to remain awake. It can result in difficulties with activities of daily living and cause patient safety issues. This includes an increased risk of injury and accidents: one questionnaire study reported that 11% of 5210 PD patients with a driving license had caused a road traffic accident and these individuals were more likely to have a higher ESS score [42]. The ESS is a convenient method of assessing daytime sleepiness, with scores ranging from 0–24 (> 10 generally considered to be pathological). In a study of 118 PD patients, Breen et al. found that 49% of PD patients had an ESS score > 10 [7]. Similar figures have been reported by other studies, with prevalence rates even higher in those PD patients with dementia [6]. EDS has been shown to predate a diagnosis of PD in some studies [1, 19]. EDS can also be assessed using the multiple sleep latency test (MSLT), with sleep latency less than 5 min considered pathological. Some PD patients experience more abrupt “sleep attacks” which have a more narcolepsy-like phenotype.

RLS and PLMS often co-exist and are highly prevalent in PD [14, 71], with RLS affecting around 30% of patients [39]. RLS is characterised by a marked urge to move (particularly the legs), typically in the evening and during periods of rest and inactivity, which is relieved by moving and only returns when the movement stops. Dopamine dysfunction, and in some cases iron deficiency, have been implicated in the pathophysiology of RLS [13, 46]. There is also a link between RLS and other comorbidities such as renal disease, type 2 diabetes mellitus, migraine and neuropsychiatric disorders [5].

PLMS are typically a repetitive limb movement, often dorsiflexion of the foot along with partial hip and knee flexion. They can occur in the general population [45] but are present in up to 87% of individuals with RLS [43].

Breathing disorders are a recognised feature of PD. In 1984, Vincken et al. used EMG and laryngoscopy to identify intermittent rhythmic or irregular flow oscillations (or even complete obstruction) of the glottis and supraglottic structures in patients with extrapyramidal disease [66]. Prevalence figures for OSA in PD are varied, with most studies being small and involving patients with co-existing sleep disorders. Cochen De Cock et al. studied 100 PD patients, 50 of whom had been referred due to EDS and 50 with no prior sleep disorder diagnosis. They found 27% of PD patients had sleep apnoea (17% mild–moderate, 10% severe) compared to 40% in matched controls [15]. On the other hand, Trotti et al. carried out overnight polysomnography on 55 patients with idiopathic PD for 3 consecutive nights and found no difference in the rates of OSA between PD patients and controls [61].

## Aetiology of sleep disorders in PD

The pathophysiology of sleep disorders in PD is likely to be multifactorial and secondary to PD nocturnal features, underlying neuronal damage, and side effects of PD medications [11]. This varied aetiology is particularly evident with insomnia, where sleep disruption can be caused by difficulty in moving in bed at night (often due to nocturnal wearing off), tremor, neuropsychiatric symptoms or pain. Whilst some of these symptoms can be remedied with symptomatic medications, some patients continue to experience sleep issues.

Changes in sleep architecture due to underlying damage to key regions involved in sleep regulation are likely to account, at least in part, for the difficulties that PD patients experience in getting to and staying asleep. Indeed, several of these brain regions (e.g. locus coeruleus, hypothalamus, amygdala, thalamus, pedunculopontine nucleus) have been shown to be affected by Lewy body pathology in post-mortem studies [30].

Alterations in hormonal rhythms have been implicated in causing PD-related sleep dysfunction. Cortisol and melatonin output are controlled by the SCN and show diurnal variation, with a cortisol peak early in the morning and a melatonin peak in the evening. It has been demonstrated that these hormonal rhythms are disrupted in PD patients (and other neurodegenerative diseases such as Alzheimer’s and Huntington’s disease). Videnovic et al. found lower levels of melatonin (reduction in amplitude and 24-h area under the curve) in 20 PD patients compared to 15 age-matched controls in a carefully controlled study taking into account light exposure, food intake and level of physical activity [62]. This difference was even more pronounced in patients with EDS. Similar findings were reported by Breen et al. who reported reduced 24-h melatonin output in 30 early stage PD patients versus 15 age-matched controls, along with a correlation between melatonin AUC and proportion of slow wave and REM sleep [8]. A subsequent study by the same group showed that hypothalamic grey matter volume was significantly reduced in PD patients and associated with decreased melatonin output, suggesting a link between degenerative changes in the hypothalamus and sleep disruption in PD [9].

Altered sleep architecture and disrupted sleep patterns may directly lead to EDS, however, there is also a link between dopaminergic medications (particularly dopamine agonists) and EDS [27, 47]. The use of dopamine agonists has been shown to contribute to worsening of ESS scores [24], as has non-tremor dominant motor phenotype and common variations in the catechol-O-methyltransferase gene involved in dopamine breakdown [7].

Hypocretin (also known as orexin) is produced by the hypothalamus and acts to promote wakefulness. Deficits in this signalling system have previously been linked to narcolepsy and cataplexy. It has also been demonstrated that there is a loss of hypocretin neurons in individuals with PD. In a study by Thannickal et al., the hypothalami of 11 PD patients and 5 healthy control patients were examined and a reduction in hypocretin neurons was seen in PD patients, with more marked reduction in advanced disease [59]. Fronczek et al. studied the brain tissue of nine late-stage PD patients and found an almost 50% reduction in total hypocretin neurons in the prefrontal cortex and hypothalamus compared to 16 control subjects, alongside reduced hypocretin concentration in the ventricular cerebrospinal fluid (CSF) [17]. Wienecke et al. studied spinal CSF hypocretin levels in ten early and treatment naive PD patients, ten advanced PD patients and ten age-matched control subjects. Whilst a downward trend in hypocretin levels was noted between early and advanced patients, no statistically significant between-group differences were found [67]. However, in two patients with advanced PD in this study, a second CSF hypocretin measurement 4–5 years later showed a reduction over time, suggesting a progressive loss of hypocretin with advancing disease.

## REM sleep behaviour disorder

REM sleep behaviour disorder (RBD) is one of the most studied sleep phenomena in PD, owing to the strong link between RBD and α-synucleinopathies. RBD occurs when individuals lose the usual muscle atonia associated with REM sleep, leading to dream enactment behaviours (which can be simple or complex movements) and/or vocalisations. The dreams experienced are usually vivid and often unpleasant or frightening. These behaviours should be confirmed by polysomnography to make a definitive RBD diagnosis.

The locus coeruleus/subcoeruleus in the brainstem, which is equivalent to the sublaterodorsal tegmental nucleus in rats, is involved in maintaining REM atonia through inhibitory projections to spinal motor neurons. Garcia et al. showed that there is a role for the glutamate neurones in the rat sublaterodorsal tegmental nucleus in generating muscle atonia in sleep [20]. When glutamate transmission was reduced through inactivation of the glutamate transporter gene *SLC17A6,* sleep-induced muscle atonia was diminished and there was an increase in motor activity during sleep. In humans, a neuromelanin-sensitive MRI study demonstrated decreased signal intensity within the locus coeruleus/subcoerulus and a corresponding increase in muscle tone during sleep in those individuals with RBD [22].

In the general population, “idiopathic” RBD has a prevalence of 0.38–1.15% [12, 31]. Over 80% of individuals with RBD go on to develop an overt α-synucleinopathy [either PD, multiple system atrophy (MSA) or dementia with Lewy bodies (DLB)], and RBD has been shown to predate the onset of motor symptoms by several decades in some individuals [53]. In other words, RBD is a strong prodromal marker of these diseases. In the largest study to date, Postuma et al. found a phenoconversion rate of 6.3% per year for individuals with idiopathic (or rather isolated) RBD [51]. A number of clinical and biological markers were identified that increased the risk of earlier phenoconversion such as older age, mild motor impairment, olfactory deficits, autonomic dysfunction (e.g. erectile dysfunction, constipation) and abnormal dopamine transporter imaging.

In the future, individuals with RBD may be an attractive patient population to test new disease-modifying drug therapies when they become available. However, it is worth noting that PD patients with RBD tend to exhibit a more severe disease phenotype with increased falls, reduced responsiveness to dopaminergic drugs and increased non-motor features (such as cognitive impairment, constipation and dizziness) [4].

Less commonly, RBD can arise in individuals with structural brain lesions (such as brainstem stroke or inflammatory lesions), Machado–Joseph disease (SCA-3), autoimmune brain diseases (such as anti-IgLON5 disease) and other pathologies. Some medications, in particular antidepressants such as selective serotonin reuptake inhibitors, have been noted to exacerbate or even trigger RBD [50]. It is unclear if RBD seen in these individuals is a pure side effect of the medication, or, more likely, if these medications unmask an underlying susceptibility in people at risk of developing brain disease in the future [50].

## Glymphatic system

The brain lacks an intrinsic lymphatic system. Instead, it depends upon the CSF, interstitial fluid and water transport (facilitated by aquaporin 4 [AQP4] channels) to clear waste proteins from the brain. Due to this reliance on glial cells, Iliff et al. termed this the “glymphatic system” [29]. They studied the accumulation of β-amyloid in the brains of healthy mice, alongside *AQP4* gene knockout mice. Where the channel was deleted, there was a marked decrease (55%) of β-amyloid clearance. A subsequent study showed the role of this system in the clearance of tau [28].

Following this, the role for sleep in the efficient functioning of the glymphatic system was demonstrated using radiolabeled β-amyloid in awake, asleep and anaesthetised mice [69]. β-amyloid was cleared twice as quickly in asleep and anaesthetised mice than in awake mice, which was reported to be due to an increase in the interstitial space during sleep, allowing for greater exchange of fluids (and thus waste proteins).

Complementary findings have now been reported in humans. A study from Shokri-Kojori et al. used positron emission tomography (PET) in 20 healthy individuals to show that one night of sleep deprivation was sufficient to increase the burden of β-amyloid in the hippocampus and the thalamus [55]. Similarly, Lucey et al. sampled CSF β-amyloid in individuals with normal sleep, induced sleep (with sodium oxybate) and sleep deprivation. This study showed a 25–30% increase in β-amyloid in sleep-deprived individuals [37]. Holth et al. sampled CSF β-amyloid, tau and α-synuclein in a group of individuals after normal sleep and 1 night of sleep deprivation and found a 30% increase in CSF β-amyloid and 36% increase in α-synuclein following sleep deprivation [26]. It is unknown whether increased production or reduced clearance of waste proteins is responsible for this.

Based on these findings, it has been proposed that the glymphatic system may play a key role in the removal of toxic proteins from the brain (particularly during sleep). Previous research has shown a decrease in the activity of the glymphatic system with advancing age [32].

## Sleep and circadian disruption as a risk factor for PD?

The discovery of the glymphatic system has added to the hypothesis that sleep and circadian disruption may cause, or accelerate, age-related brain diseases. Other mediators of the relationship between sleep and brain health may include oxidative stress, inflammation, blood brain–barrier integrity and biological ageing. Prospective studies have demonstrated a link between sleep disruption and incident cognitive impairment [70], but there is now emerging evidence related to PD.

A recent study by Sohail et al. suggested that sleep disturbances other than RBD may predate the onset of PD motor symptoms. 269 older adults without PD were assessed for sleep fragmentation during their life (using wrist-worn actigraphy recording for 7 consecutive days) and followed up until death. The median time between actigraphy data and death was 1.4 years. The study found that individuals with sleep fragmentation had a higher presence of Lewy body pathology and substantia nigra cell loss on post-mortem examination [56].

Lysen et al. prospectively assessed subjective sleep quality as part of a nested cohort study of 7726 individuals within the Rotterdam study. Poor sleep quality and shorter duration of sleep were associated with an increased risk of parkinsonism and PD over the next 2 years, with the strength of this relationship attenuating over longer follow-up. Like the study from Sohail et al., the authors concluded that sleep disturbances may be a prodromal feature of PD rather than a causal factor per se [38], however further research involving large cohorts and a range of sleep (both subjective and objective) and brain health measures are needed.

In a recent study by Noyce et al., mendelian randomisation was used to highlight a potential causal association between being a “morning person” and subsequent increased risk of developing PD [44], although the precise mechanisms behind this are not clear.

## Therapeutic approaches

Management of sleep disorders should begin with simple measures, aiming for optimum management of motor symptoms and ensuring good sleep hygiene [64]. These practices include advice on regularising bed times and light exposure, avoiding wake-promoting substances (such as nicotine and caffeine) and medications later in the day, avoiding prolonged time in bed whilst awake, and avoiding exercising later in the day [58, 72].

Given the blunted melatonin rhythms observed in PD patients, melatonin supplementation should be considered for the treatment of insomnia. Dowling et al. assessed the efficacy of melatonin administration for sleep disturbances in 40 PD patients [16]. Although no significant differences were seen when 5 mg melatonin was compared to placebo, a small (but statistically significant) increase in sleep time was noted with 50 mg melatonin. In a similar study, Medeiros et al. randomised 18 patients to receive either 3 mg melatonin or placebo [41]. Again, no significant differences were found between the groups. Whilst these studies did not report substantial objective benefit from melatonin therapy, patients reported subjective benefits. It should be noted that these were relatively small studies of heterogenous populations with a mixture of sleep disturbances.

Current treatment options for RBD involve supportive management including altering the sleeping environment to make it safer (e.g. moving furniture and placing cushions next to the bed). Melatonin and clonazepam are the mainstay of pharmacological management [33, 35], although a recent study found no overall benefit in RBD symptoms using prolonged release melatonin compared to placebo [23].

Management of RLS includes iron replacement in individuals with proven iron deficiency and avoidance of medications known to exacerbate RLS (such as antidepressants, antihistamines and dopamine antagonists). If additional treatment is required, dopamine agonists are commonly started, with consideration given to the possibility of augmentation (a poorly understood phenomenon that occurs with long-term dopaminergic therapy characterised by more severe symptoms occurring earlier in the day). Alpha-2 delta ligands (such as pregabalin) are now considered an alternative first-line treatment [2], but the initial drug of choice should be carefully chosen according to symptomatology and the individual needs [21, 68].

Part of the control of circadian rhythmicity comes from input from specialised retinal ganglion cells that receive light input and assist in entraining the sleep–wake cycle. These pathways have been shown to be damaged in PD, with evidence of dopaminergic retinal degeneration [25]. Studies have also indicated that individuals with PD are exposed to less natural light throughout the day, presumably due to the impact of their disease on daily functioning [57]. It is also likely that even in PD patients receiving normal levels of natural light, damage to the SCN may affect the central circadian pacemaker. To counteract this, it has been proposed that light therapy could be beneficial. Videnovic et al. studied the effect of bright light therapy (10,000 lx) compared to a control group of dim-red light (< 300 lx) in PD patients. Light therapy was delivered in two 1-h sessions every day for 2 weeks, resulting in self-reported benefits in sleep quality, ease of falling asleep and improved disease severity (as assessed using the Unified Parkinson’s Disease Rating Scale (UPDRS)). There was also decreased sleep latency and an overall increase in physical activity on actigraphy monitoring. Some benefits were noted in the dim-red light therapy group as well, which was suggested to be related to the daily structure imposed by the regular light therapy [65]. Martino et al. carried out a retrospective study on individuals with PD who had received timed light therapy (3000–4000 lx) for a 1-h period 1–4 h before sleep [40]. Light therapy improved insomnia and reduced nocturnal movement. These studies have proven that light therapy is safe and feasible to deliver. Larger studies are now required to prove its therapeutic efficacy, as well as decide upon optimum timings and intensities of light therapy delivery.

Finally, the orexin signalling system is a promising treatment target for some PD-related sleep disorders. The dual orexin receptor antagonist suvorexant has been approved as a treatment for primary insomnia. Studies are underway to assess its benefit in neuropsychiatric and neurodegenerative disorders including Parkinson’s disease (ClinicalTrials.gov Identifier: NCT02729714).

## Conclusion

Sleep is of great interest in the field of PD and neurodegenerative diseases for a number of reasons. First, it is a common symptom that has an impact on quality of life and can influence daytime functions (including cognitive abilities). Second, there are some aspects of sleep (such as RBD) which may provide a window into the early prodromal period and enable potentially therapeutic interventions to target this patient population. Third, there is emerging evidence that sleep directly influences brain health and could even be a risk factor for the development and progression of age-related brain diseases. By understanding the full spectrum of sleep disorders and their causes, we will hopefully be able to improve quality of life in patients and potentially influence the course of their disease.

## References

[1] Abbott RD, Ross GW, White LR (2005). Excessive daytime sleepiness and subsequent development of Parkinson disease. Neurology.

[2] Allen RP, Chen C, Garcia-Borreguero D (2014). Comparison of pregabalin with pramipexole for restless legs syndrome. N Engl J Med.

[3] Barone P, Antonini A, Colosimo C (2009). The Priamo study: a multicenter assessment of nonmotor symptoms and their impact on quality of life in Parkinson’s disease. Mov Disord.

[4] Barber TR, Lawton M, Rolinski M (2017). Prodromal Parkinsonism and neurodegenerative risk stratification in REM sleep behaviour disorder. Sleep.

[5] Becker PM, Novak M (2014). Diagnosis, comorbidities, and management of restless legs syndrome. Curr Med Res Opin.

[6] Boddy F, Rowan EN, Lett D (2007). Subjectively reported sleep quality and excessive daytime somnolence in Parkinson's disease with and without dementia, dementia with Lewy bodies and Alzheimer's disease. Int J Geriatr Psychiatry.

[7] Breen DP, Williams-Gray CH, Mason SL (2012). Excessive daytime sleepiness and its risk factors in incident Parkinson's disease. J Neurol Neurosurg Psychiatry.

[8] Breen DP, Vuono R, Nawarathna U (2014). Sleep and circadian rhythm regulation in early Parkinson disease. JAMA Neurol.

[9] Breen DP, Nombela C, Vuono R (2016). Hypothalamic volume loss. Is associated with reduced melatonin output in Parkinson’s disease. Mov Disord.

[10] Camacho-Soto A, Warden MN, Searles Nielsen S (2017). Traumatic brain injury in the prodromal period of Parkinson’s disease: a large epidemiological study using Medicare data. Ann Neurol.

[11] Chaudhuri KR, Schapira AH (2009). Non-motor symptoms of Parkinson’s disease: dopaminergic pathophysiology and treatment. Lancet Neurol.

[12] Chiu HF, Wing YK, Lam LC (2000). Sleep-related injury in the elderly: an epidemiological study in Hong Kong. Sleep.

[13] Connor JR, Wang ZS, Allen RP (2009). Altered dopaminergic profile in the putamen and substantia nigra in restless leg syndrome. Brain.

[14] Covassin N, Neikrug AB, Liu L (2012). Clinical correlates of periodic limb movements in sleep in Parkinson’s disease. J Neurol Sci.

[15] Cochen De Cock VC, Abouda M, Leu S (2010). Is obstructive sleep apnea a problem in Parkinson’s disease?. Sleep Med.

[16] Dowling GA, Mastick J, Colling E (2005). Melatonin for sleep disturbances in Parkinson’s disease. Sleep Med.

[17] Fronczek R, Overeem S, Lee SY (2007). Hypocretin (orexin) loss in Parkinson’s disease. Brain.

[18] Gallagher DA, Lees AJ, Schrag A (2010). What are the most important nonmotor symptoms in patients with Parkinson’s disease and why are we missing them?. Mov Disord.

[19] Gao J, Huang X, Park Y (2011). Daytime napping, nighttime sleeping and Parkinson disease. Am J Epidemiol.

[20] Garcia SV, Libourel PA, Lazarus M (2017). Genetic inactivation of glutamate neurons in the rat sublaterodorsal tegmental nucleus recapitulates REM sleep behaviour disorder. Brain.

[21] Garcia-Borreguero D, Silber MH, Winkelman JW (2016). Guidelines for the first-line treatment of restless legs syndrome/Willis-Ekbom disease, prevention and treatment of dopaminergic augmentation: a combined task force of the IRLSSG, EURLSSG, and the RLS Foundation. Sleep Med.

[22] Garcia-Lorenzo D, Longo-Dos Santos C, Ewencyzk C (2013). The coeruleus/subcoeruleus complex in rapid eye movement sleep behaviour disorders in Parkinson’s disease. Brain.

[23] Gilat M, Jackson AC, Marshall NS (2019). Melatonin for rapid eye movement sleep behaviour disorder in Parkinson’s disease: a randomised controlled trial. Mov Disord.

[24] Gjerstad MD, Alves G, Wentzel-Larsen T (2006). Excessive daytime sleepiness in Parkinson disease. Is it the drugs or the disease?. Neurology.

[25] Harnois C, Di Paolo T (1990). Decreased dopamine in the retinas of patients with Parkinson’s disease. Invest Ophthalmol Vis Sci.

[26] Holth JK, Fritschi SK, Wang C (2019). The sleep-wake cycle regulates brain interstitial fluid tau in mice and CSF tau in humans. Science.

[27] Homann CN, Wenzel K, Suppan K (2002). Sleep attacks in patients taking dopamine agonists. BMJ.

[28] Iliff JJ, Chen MJ, Plog BA (2014). Impairment of glymphatic pathway function promotes tau pathology after traumatic brain injury. J Neurosci.

[29] Iliff JJ, Wang M, Liao Y (2012). A paravascular pathway facilitates CSF flow through the brain parenchyma and the clearance of interstitial Solutes, including amyloid beta. Sci Transl Med.

[30] Kalaitzakis ME, Gentleman SM, Pearce RKB (2013). Disturbed sleep in Parkinson’s disease: anatomical and pathological correlates. Neuropathol Appl Neurobiol.

[31] Kang S-H, Yoon IT, Lee SD (2013). REM sleep behaviour disorder in the Korean elderly population: prevalence and clinical characteristics. Sleep.

[32] Kress BA, Iliff JJ, Xia M (2014). Impairment of paravascular clearance pathways in the aging brain. Ann Neurol.

[33] Kunz D, Mahlberg R (2010). A two-part, double-blind, placebo-controlled trial of exogenous melatonin in REM sleep behaviour disorder. J Sleep Res.

[34] Kurtis MM, Balestrino R, Rodriguez-Blazquez C (2018). A review of scales to evaluate sleep disturbances in movement disorders. Front Neurol.

[35] Li SX, Lam SP, Zhang J (2016). A prospective, naturalistic follow-up study of treatment outcomes with clonazepam in rapid eye movement sleep behaviour disorder. Sleep Med.

[36] Li W, Ma L, Yang G, Gan W-B (2017). REM sleep selectively prunes and maintains new synapses in development and learning. Nat Neurosci.

[37] Lucey BP, Hicks TJ, McLeland JS (2018). Effect of sleep on overnight cerebrospinal fluid amyloid beta kinetics. Ann Neurol.

[38] Lysen TS, Darweesh SKL, Ikram MK (2019). Sleep and risk of parkinsonism and Parkinson’s disease: a population-based study. Brain.

[39] Martinez-Martin P, Rodriguez-Blazquez C, Kurtis MM (2011). The impact of non-motor symptoms on health-related quality of life of patients with Parkinson’s disease. Mov Disord.

[40] Martino JK, Freelance CB, Willis GL (2018). The effect of light exposure on insomnia and nocturnal movement in Parkinson’s disease: an open label, retrospective, longitudinal study. Sleep Med.

[41] Medeiros CA, Carvalhedo de Bruin PF, Lopes LA (2007). Effect of exogenous melatonin on sleep and motor dysfunction in Parkinson’s disease. A randomized, double blind, placebo-controlled study. J Neurol.

[42] Meindorfner C, Komer Y, Moller JC (2005). Driving in Parkinson’s disease: mobility, accidents, and sudden onset of sleep at the wheel. Mov Disord.

[43] Montplaisir J, Boucher S, Poirier G (1997). Clinical, polysomnographic and genetic characteristics of restless legs syndrome: a study of 133 patients diagnosed with new standard criteria. Mov Disord.

[44] Noyce AJ, Kia DA, Heilbron K (2018). Tendency towards being a “Morning person” increases the risk of Parkinson’s disease: evidence from Mendelian randomisation. BioRxiv.

[45] Ohayon and Roth (2002). Prevalence of restless legs syndrome and periodic limb movement disorder in the general population. J Psychosom Res.

[46] Ondo WG, Vuong KD, Jankovic J (2002). Exploring the relationship between Parkinson disease and restless legs syndrome. Arch Neurol.

[47] Paus S, Brecht HM, Koster J (2003). Sleep attacks, daytime sleepiness, and dopamine agonists in Parkinson’s disease. Mov Disord.

[48] Peeraully T, Yong MH, Chokroverty S, Tan EK (2012). Sleep and Parkinson’s disease: a review of case–control polysomnography studies. Mov Disord.

[49] Poewe W (2017). Parkinson disease. Nat Rev Dis Primers.

[50] Postuma RB, Gagnon JF, Tuineaig M (2013). Antidepressants and REM sleep behaviour disorder: isolated side effect or neurodegenerative signal?. Sleep.

[51] Postuma RB, Iranzo A, Hu M (2019). Risk and predictors of dementia and parkinsonism in idiopathic REM sleep behaviour disorder: a multi centre study. Brain.

[52] Raven F, Van der Zee EA, Meerlo P, Havekes R (2018). The role of sleep in regulating structural plasticity and synaptic strength: Implications for memory and cognitive function. Sleep Med Rev.

[53] Schenk CH, Boeve BF, Mahowald MW (2013). Delayed emergence of a parkinsonism disorder or dementia in 81% of older men initially diagnosed with idiopathic rapid eye movement sleep behaviour disorder: a 16-year update on a previously reported series. Sleep Med.

[54] Seppi K, Ray Chaudhuri K, Coelho M (2019). Update on treatments for nonmotor symptoms of Parkinson’s disease—an evidence based medicine review. Mov Disord.

[55] Shokri-Kojiri E, Wang G-J, Wiers CE (2018). β-Amyloid accumulation in the human brain after one night of sleep deprivation. PNAS.

[56] Sohail S, Yu L, Schneider JA (2017). Sleep fragmentation and Parkinson’s disease pathology in older adults without Parkinson’s disease. Mov Disord.

[57] Speelman AD, van de Warrenburg BP, van Nimwegen M (2011). How might physical activity benefit patients with Parkinson disease?. Nat Rev Neurol.

[58] Stepanski EJ, Wyatt JK (2003). Use of sleep hygiene in the treatment of insomnia. Sleep Med Rev.

[59] Thannickal TC, Lai YY, Siegel JM (2007). Hypocretin (orexin) cell loss in Parkinson’s disease. Brain.

[60] Tononi G, Cirelli C (2014). Sleep and the price of plasticity: from synaptic and cellular homeostasis to memory consolidation and integration. Neuron.

[61] Trotti LM, Bliwise DL (2010). No increased risk of obstructive sleep apnea in Parkinson’s disease. Mov Disord.

[62] Videnovic A, Noble C, Reid KJ (2014). Circadian melatonin rhythm and excessive daytime sleepiness in Parkinson’s disease. JAMA Neurol.

[63] Videnovic A, Lazar AS, Barker RA, Overeem S (2014). “The clocks that time us”—circadian rhythms in neurodegenerative disorders. Nat Rev Neurol.

[64] Videnovic A (2017). Management of sleep disorders in Parkinson’s disease and multiple system atrophy. Mov Disord.

[65] Videnovic A, Klerman ED, Wang W (2017). Timed light therapy for sleep and daytime sleepiness associated with Parkinson disease: a randomized clinical trial. JAMA Neurol.

[66] Vincken WG, Gauthier SG, Dollfuss RE (1984). Involvement of upper-airway muscles in extrapyramidal disorders—a cause of airflow limitation. New Engl J Med.

[67] Wienecke M, Werth E, Poryazova R (2012). Progressive dopamine and hypocretin deficiencies in Parkinson’s disease: is there an impact on sleep and wakefulness?. J Sleep Res.

[68] Winkelman JW, Armstrong MJ, Allen RP (2016). Practice guideline summary: treatment of restless legs syndrome in adults: report of the guideline development, dissemination, and implementation subcommittee of the American Academy of Neurology. Neurology.

[69] Xie L, Kang H, Xu Q (2013). Sleep drives metabolite clearance from the adult brain. Science.

[70] Yaffe K, Falvey CM, Hoang T (2014). Connections between sleep and cognition in older adults. Lancet Neurol.

[71] Yang X, Liu B, Shen H (2018). Prevalence of restless legs syndrome in Parkinson’s disease: a systematic review and meta-analysis of observational studies. Sleep Med.

[72] Youngstedt SD, O’Connor PJ, Dishman RK (1997). The effects of acute exercise on sleep: a quantitative synthesis. Sleep.

